# Effective Mechanical Insufflation-Exsufflation in a Patient With Difficulty in Sputum Discharge and Intensive Care Unit-Acquired Weakness: A Case Report

**DOI:** 10.7759/cureus.21847

**Published:** 2022-02-02

**Authors:** Tadayoshi Nonoyama, Hiroko Shigemi, Chiaki Yasutake, Akihiko Matsumine, Tamotsu Ishizuka

**Affiliations:** 1 Department of Rehabilitation, University of Fukui Hospital, Fukui, JPN; 2 Respiratory Medicine, University of Fukui, Fukui, JPN; 3 Faculty of Medical Sciences, Kyoto Prefectural University of Medicine, Kyoto, JPN; 4 Orthopedics and Rehabilitation Medicine, University of Fukui, Fukui, JPN

**Keywords:** musculoskeletal rehabilitation, respiratory care, mechanical insufflation-exsufflation, icu-acquired weakness, intensive care unit

## Abstract

Intensive care unit-acquired weakness (ICU-AW), a common complication in critically ill patients, may result in diaphragmatic dysfunction, which delays weaning from artificial ventilators. Here, we present the case of a patient with difficulty in sputum discharge due to ICU-AW. In the ICU, postural drainage sputum aspiration by bronchoscopy and squeezing were performed daily, but the patient’s condition did not resolve. Mechanical insufflation-exsufflation (MI-E) enabled the sputum to move to the main bronchus from the peripheral bronchi, and suctioning using a bronchoscope was no longer necessary. However, the presence of sputum persisted, and MI-E was necessary after weaning, proving crucial in treating the patient with sputum discharge difficulty complicated by ICU-AW after being removed from an artificial ventilator. MI-E can be useful for patients with difficulty in sputum discharge due to ICU-AW; however, the weaning process may be prolonged in such cases.

## Introduction

Intensive care unit-acquired weakness (ICU-AW) is a neuromuscular complication that results in symmetrical limb muscle weakness after prolonged admission to the ICU, occurring in about 50% of critically ill patients with sepsis, multiple organ failure, or long-term ventilatory support [1]. In patients with ICU-AW, 80% develop diaphragmatic dysfunction which delays the weaning process from artificial ventilators [2,3]. Therefore, patients with ICU-AW may need respiratory physiotherapy to treat any difficulties in expectorating.

Mechanical insufflation-exsufflation (MI-E) increases expiratory flow velocity by applying positive pressure to the airway and then rapidly switching to negative pressure to promote expulsion of airway secretions and maintain airway clearance. The use of MI-E has been reported to increase sputum output in patients with reduced coughing ability [4]. However, regarding ICU-AW cases, there are no reports on the use of MI-E for airway clearance. Moreover, there are no case reports of ICU patients requiring MI-E for a prolonged period, such as after ICU discharge or after discharge from an acute hospital.

In this case report, we describe a patient with sepsis complicated by ICU-AW and difficulty with sputum discharge. Although the introduction of MI-E was effective, it was difficult to wean the patient, and the intervention was prolonged. This is the first report to show the effectiveness of MI-E for ICU-AW.

## Case presentation

**Figure 1:**
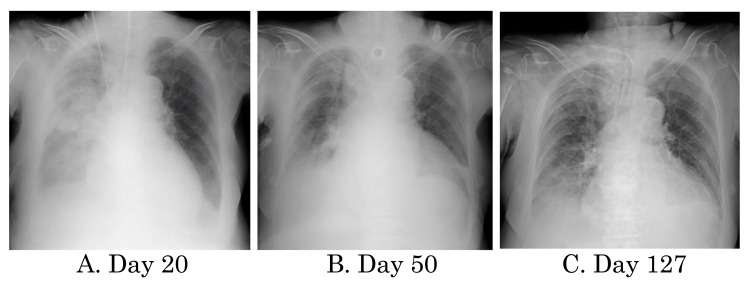
Chest X-rays on hospital admission day 20 (A), day 50 (B), and day 127 (C).

The patient is a 76-year-old woman with no special family history. She was being treated with prednisolone 5 mg/day, non-steroidal anti-inflammatory drugs, and biologics (etanercept) since the age of 61 for rheumatoid arthritis. The patient was admitted to our hospital for an iliolumbar abscess and sepsis, probably due to excessive immunosuppression caused by a self-administered overdose of biologics. Twenty days after admission, the patient was admitted to the ICU for right aspiration pneumonia, asphyxia due to sputum, and acute respiratory failure. On the same day, the patient was intubated and required ventilatory management (pressure-controlled ventilation set at FiO_2_ 0.5, inspiratory pressure 17 cmH_2_O, and positive end-expiratory pressure (PEEP) 8 cmH_2_O). Chest X-ray showed pleural effusion with atelectasis and decreased permeability of the right lung field, and a right thoracic drain was inserted (Figure 1, Panel A). The patient had a left ventricular ejection fraction of 40%, heart rate of 100-110 beats per minute, and blood pressure of 150-160/60-80 mmHg. She was treated with dobutamine, carperitide, and furosemide. Respiratory rehabilitation by a physical therapist was started on day 21.

**Figure 2:**
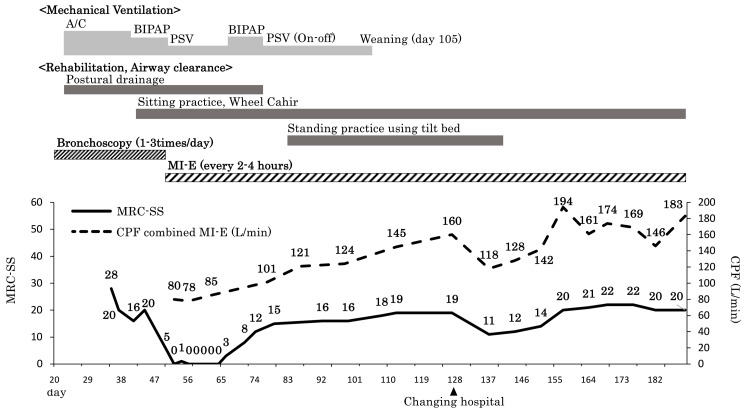
Clinical course. A/C: assist/control; BIPAP: biphasic positive airway pressure; PSV: positive airway pressure; MI-E: mechanical insufflation-exsufflation; MRC-SS: Medical Research Council-sum score; CPF: cough peak flow

The patient’s clinical course is shown in Figure 2. Because the patient had a lot of airway sputum and required frequent endotracheal suctioning, we started positional drainage and squeezing. However, a decrease in SpO_2_ was observed due to sputum obstruction in the central airway, and it was difficult to improve the condition with endotracheal suction alone. The bronchoscopic findings showed viscous sputum spurting from the bilateral peripheral airways, and it was difficult to completely remove the sputum by positional drainage, squeezing, or endotracheal suction. Therefore, sputum collection by bronchoscopy was initiated at a frequency of one to three times a day. At this point, the introduction of MI-E was considered. Because the chest X-ray showed pleural effusion and the PEEP was 10 cmH_2_O, we were concerned about alveolar collapse due to the release of positive pressure if MI-E was performed and decided not to introduce it. A tracheostomy was performed on day 30. Sedation level was managed using the Richmond Agitation-Sedation Scale (-1 to 0), and limb muscle strength was assessed with the Medical Research Council-sum score (MRC-SS), which was 28 points on day 35 and 5 points on day 50. There were no abnormal findings on head computed tomography or magnetic resonance imaging. The diagnostic criteria for ICU-AW are as follows: (1) generalized weakness occurring after the onset of critical illness; (2) diffuse weakness (involving both proximal and distal muscles), symmetric, flaccid, and generally not involving the cranial nerves; (2) MRC-SS of <48 of all muscle groups that can be tested at least twice in 24 hours; (4) dependence on ventilation; and (5) exclusion of causes of weakness that are not related to underlying serious diseases [5]. The patient had severe muscle weakness that met all the above criteria.

On day 50, the patient’s difficulty in expectorating persisted, and bronchoscopic sputum spurting was performed daily. The ventilator mode could be changed to pressure support ventilation (FiO_2_ 0.5, pressure support 7 cmH_2_O, and PEEP 5 cmH_2_0), and the chest X-ray showed a decrease in pleural effusion (Figure 1, Panel B). Hence, MI-E (CoughAssist E70, Philips Respironics, Murrysville, PA, USA) was introduced due to the patient’s difficulty with expectoration. The machine was set at inspiratory pressure 40 hPa for 3.0 seconds, expiratory pressure -40 hPa for 2.5 seconds, and oscillation mode 13 Hz ± 8 hPa. After initiation of MI-E, a large amount of yellow viscous sputum was collected for the first time, and her cough peak flow (CPF) was 80-90 L/minute. Because sputum transfer to the central airway became possible, sputum collection by bronchoscopy became unnecessary and the intervention was discontinued.

For rehabilitation of the patient, dynamic joint exercises, muscle stretching of the extremities, and neuromuscular electrical stimulation for lower extremities were started from the day after admission to the ICU. Mobilization was initiated from day 42, and dynamic sitting exercises and wheelchair sitting practice were performed. However, the patient’s muscle weakness progressed resulting in an MRC-SS of 0 and Barthel index score of 0 on day 52 of admission. The patient was discharged from the ICU to a rehabilitation unit on day 56 and weaned from the ventilator on day 105. However, her sputum expectorant capacity did not improve, and the MI-E was re-initiated. Her MRC-SS at day 109 was 18 and her CPF with MI-E was 140-160 L/min. An evaluation of her swallowing function showed salivary retention in the piriform fossa, suggesting a chronic increase in airway sputum due to subclinical aspiration. The MRC-SS and CPF started to improve, but the patient continued to have difficulty in discharging sputum on her own, and MI-E was continued.

On day 96, transfer coordination of the patient to a different facility was initiated. Because the patient needed to continue MI-E, many hospitals were not equipped to accept her and it was difficult to find a hospital for transfer. The patient’s chest X-ray on day 127 showed that her lungs and permeability had improved (Figure 1, Panel C), and she was transferred to a distant hospital that offered MI-E support on day 128. Although the patient’s activities of daily living did not improve at the new hospital (Barthel index score 0 points), her MRC-SS (20 points), CPF (183 L/minute), and limb and respiratory muscle strength tended to improve. Thus, the frequency of MI-E intervention was gradually decreased and completely discontinued on day 191 when airway clearance could be maintained by endotracheal suction alone.

## Discussion

This report aimed to show the effectiveness of MI-E for ICU-AW in a patient with sepsis. Two important clinical points are shown in this case. First, MI-E can be effective for impaired airway clearance in cases of ICU-AW. In addition, the MI-E weaning process in severe ICU-AW cases may be prolonged.

In the present case, MI-E was effective for impaired airway clearance in a patient with ICU-AW. Because ICU-AW is diagnosed by assessing the muscle strength of the extremities, there is great uncertainty regarding respiratory impairment. It has been reported that diaphragm muscle fibers atrophy by more than 50% after 18-69 hours of ventilation [6]. Because our patient was under long-term ventilatory management, diaphragm atrophy may have been a considerable factor. However, evidence regarding the comorbidity of diaphragmatic dysfunction and ICU-AW is limited [7]. Sputum dysfunction may be a separate pathology from ICU-AW. The introduction of MI-E before and after extubation for acute respiratory failure in ICU patients has been shown to decrease the length of ICU stay and rates of reintubation [8]. In the present case, a tracheostomy was performed, but the patient was weaned from the ventilator because airway clearance could be maintained with MI-E. Studies suggest that MI-E is useful for expectorant support in cases of neuromuscular diseases and spinal cord injuries [9-11]. When expectorant failure is due to respiratory muscle weakness and paralysis, we believe that MI-E is an effective intervention and can be applied to respiratory failure in patients with ICU-AW because of a similar condition.

In this case, the weaning process from MI-E was prolonged. It has been reported that motor function and respiratory muscle weakness in patients with ICU-AW may be persistent, and severe cases with low MRC-SS are associated with prolonged ventilatory management [2,12]. When airway clearance is impaired, MI-E should be introduced; however, in Japan, the use of MI-E during hospitalization is not covered by medical insurance. In addition, MI-E can be used with a ventilator at home for individuals with neuromuscular diseases and spinal cord injuries, but ICU-AW is still not covered by insurance. To improve patient outcomes and quality of life, it is necessary to develop an environment in which MI-E can be used for ICU-AW patients with sputum disorders from the acute stage to home without deterrents such as cost.

## Conclusions

ICU-AW can cause difficulty in sputum discharge. In this case, the introduction of MI-E allowed us to terminate sputum removal by bronchoscopy and was effective in maintaining airway clearance; however, it took a long time to wean the patient from MI-E. MI-E may be a good indication for sputum discharge in ICU-AW patients. However, in severe cases, withdrawal of MI-E may be prolonged due to delayed muscle recovery. We recommend establishing conditions in which MI-E can be used for ICU-AW patients with sputum disorders from the acute phase to home.
